# Supplementary material for: Empirical methods for controlling false positives and estimating confidence in ChIP-Seq peaks
Source: BMC Bioinformatics. 2008 Dec 5;9:523. doi: 10.1186/1471-2105-9-523 (PMC2628906; doi:10.1186/1471-2105-9-523)
Supplement: Additional File 1 — A variety of html documents from the USeq web site detailing the available applications, their best usage, output file type descriptions, command line menus, etc. [file 1471-2105-9-523-S1.zip › USeqUserGuides/JavaDoc.zip!/JavaDoc/util/bio/annotation/ExportIntergenicRegions.html]

ExportIntergenicRegions


|  |  |  |
| --- | --- | --- |
| **Overview** \| **Package** \| **Class** \| **Use** \| **Tree** \| **Deprecated** \| **Index** \| **Help** | |  |
| **PREV CLASS**   **NEXT CLASS** | **FRAMES**    **NO FRAMES**     **All Classes** |  |
| SUMMARY: NESTED \| FIELD \| CONSTR \| METHOD | DETAIL: FIELD \| CONSTR \| METHOD |  |


---


## util.bio.annotation Class ExportIntergenicRegions

```
java.lang.Object
  util.bio.annotation.ExportIntergenicRegions
```

---

``` public class ExportIntergenicRegions extends java.lang.Object ```

---

**Constructor Summary**

`ExportIntergenicRegions(java.lang.String[] args)`


| **Method Summary** | |
| --- | --- |
| `void` | `exportIntergenicRegions()` |
| `static int[][]` | `fetchFalseBlocks(boolean[] b, int bpToTrim, int minSize)` |
| `static void` | `main(java.lang.String[] args)` |
| `boolean[]` | `maskBoolean(Gff3Feature[] features)`             Uses a boolean[] to score whether a base is part of an annotation (true) or not (false). |
| `boolean` | `printBlocks(int[][] startStop, java.lang.String chromosome, java.io.File file)`             Prints a file containing the block regions. |
| `static void` | `printDocs()` |
| `void` | `processArgs(java.lang.String[] args)`             This method will process each argument and assign new varibles |

**Methods inherited from class java.lang.Object**

`equals, getClass, hashCode, notify, notifyAll, toString, wait, wait, wait`

**Constructor Detail**

### ExportIntergenicRegions

```
public ExportIntergenicRegions(java.lang.String[] args)
```


**Method Detail**

### exportIntergenicRegions

```
public void exportIntergenicRegions()
```

---


### printBlocks

```
public boolean printBlocks(int[][] startStop,
                           java.lang.String chromosome,
                           java.io.File file)
```

:   Prints a file containing the block regions. Strips off the extension from the file and appends the chromosome name.

---


### fetchFalseBlocks

```
public static int[][] fetchFalseBlocks(boolean[] b,
                                       int bpToTrim,
                                       int minSize)
```

---


### maskBoolean

```
public boolean[] maskBoolean(Gff3Feature[] features)
```

:   Uses a boolean[] to score whether a base is part of an annotation (true) or not (false).

---


### main

```
public static void main(java.lang.String[] args)
```

---


### processArgs

```
public void processArgs(java.lang.String[] args)
```

:   This method will process each argument and assign new varibles

---


### printDocs

```
public static void printDocs()
```


---


|  |  |  |
| --- | --- | --- |
| **Overview** \| **Package** \| **Class** \| **Use** \| **Tree** \| **Deprecated** \| **Index** \| **Help** | |  |
| **PREV CLASS**   **NEXT CLASS** | **FRAMES**    **NO FRAMES**     **All Classes** |  |
| SUMMARY: NESTED \| FIELD \| CONSTR \| METHOD | DETAIL: FIELD \| CONSTR \| METHOD |  |


---
